# The biomechanical changes of facet joint violation after transforaminal lumbar interbody fusion combined with decompression surgery: a finite element study

**DOI:** 10.3389/fbioe.2025.1481719

**Published:** 2025-08-01

**Authors:** Xing Chen, Geng Zhao, Xiaoxiong Wang, Yuchen Zhang, Junyuan Sun, Xu Zhang, Xinyu Liu

**Affiliations:** ^1^Department of Orthopedics, Qilu Hospital, Cheeloo College of Medicine, Shandong University, Jinan, China; ^2^School of Instrumentation and Optoelectronic Engineering, Beihang University, Beijing, China; ^3^ University of Health and Rehabilitation Sciences, Qingdao, China; ^4^ Hefei National Laboratory, Hefei, China

**Keywords:** adjacent segment degeneration, transforaminal lumbar interbody fusion, decompression, facet joint violation, finite element

## Abstract

**Introduction:**

Facet joint violation (FJV) is a common complication of intervertebral fusion surgery, altering the load-bearing capability of the facet joints and ultimately contributing to segmental instability. Furthermore, adjacent segment degeneration is one of the potential long-term complications following lumbar spinal intervertebral fusion. For patients with a history of lumbar intervertebral fusion who developed symptomatic spinal stenosis at adjacent segments, adjacent segment decompression surgery is a clinically viable option. The biomechanical effects of isolated decompression surgery or intervertebral fusion surgery have been relatively well established. However, the biomechanical impact of facet joint intrusion on patients who have undergone both lumbar intervertebral fusion and adjacent segment decompression remains unclear.

**Methods:**

The L4-L5 intervertebral fusion model (F) and the L3-L4 decompression with L4-L5 intervertebral fusion model (DF) were developed based on a validated intact L3-L5 model (I). On the basis of DF model, six FJV models were created according to the extent and grades of facet joint violation: left mild violation (LMV), left severe violation (LSV), right mild violation (RMV), right severe violation (RSV), bilateral mild violation (BMV), and bilateral severe violation (BSV). In each scenario, the range of motion (ROM) and intradiscal pressure (IDP) at the supra-adjacent segments were analyzed.

**Results:**

The results indicated that both decompression and intervertebral fusion surgeries increased the ROM and intradiscal stress on the L3-L4 intervertebral discs. Additionally, the presence of facet joint violation further increased the ROM and intradiscal pressure on the L3-L4 segment, with these changes being associated with the grades and extent of facet joint violation, particularly when decompression and violation occurred on the same side.

**Discussion:**

This study revealed that decompression or facet joint violation could elevate intradiscal pressure and ROM at the supra-adjacent segment, indicating a potential synergistic interaction between these two risk factors.

## 1 Introduction

The intervertebral fusion surgery is a common procedure for managing degenerative lumbar spinal disorders, including spinal stenosis, spondylolisthesis, scoliosis, and multi-segment degeneration, achieving high intervertebral fusion rates and satisfactory long-term clinical outcomes (Hilibrand and Robbins, 2004; Reid et al., 2019). However, a significant complication observed after intervertebral fusion surgery is adjacent segment disease (ASD), defined as symptomatic degeneration adjacent to the fused segment (Wang et al., 2019; Zhong et al., 2017). Many risk factors associated with the development of ASD have been reported, including age, gender, high body mass index, pre-existing spinal stenosis, disc degeneration or herniation, osteophyte formation, spondylolisthesis, altered pelvic parameters or PI-LL mismatch (Mesregah et al., 2022). Besides, biomechanical studies have indicated that increases in the range of motion (ROM) and intravertebral disc pressure (IDP) at adjacent segments are primary factors that accelerate degenerative changes, thereby inducing clinical symptoms (Jiang and Li, 2019; Kim et al., 2012; Wang et al., 2019).

The reported incidence of ASD following lumbar intervertebral fusion ranges from 4.7% to 27.4% (Bae et al., 2010; Lee et al., 2014; Sato et al., 2015), and about 1/3 of them progress to clinical disease (Hashimoto et al., 2019). A multitude of factors are associated with the onset and progression of adjacent segment disease (ASD), including high body mass index (BMI), pre-existing degenerative changes, sagittal plane malalignment, concurrent decompression of adjacent segments, and facet joint violation (FJV) (Kim et al., 2011; 2016; Lai et al., 2004; Maragkos et al., 2020; Mesregah et al., 2022). Among these, FJV is the most significantly associated surgical risk factor, leading to biomechanical alterations such as abnormal facet joint loading and motion, which can accelerate the development of postoperative low back pain. Biomechanical studies have demonstrated that FJV results in increased contact forces within the facet joints and elevated intradiscal pressure in adjacent segments. When rotational forces are applied, FJV increases the range of motion in adjacent segments. Facet joint injury is associated with alterations in spinal stability and load-bearing capacity, which may ultimately contribute to the development of ASD (Hiyama et al., 2020).

Controversy persists regarding the surgical treatment of two-segment or multi-segment degenerative disorders, such as lumbar spinal stenosis adjacent to spondylolisthesis or multi-segment lumbar spinal stenosis combined with single-segment degenerative spondylolisthesis (MLSS) (Smorgick et al., 2013; Sun et al., 2019). Decompression surgery is typically recommended for lumbar spinal stenosis without accompanying instability. Despite satisfactory clinical outcomes (Amundsen et al., 2000), several studies have indicated that patients undergoing decompression surgery alone are at risk for iatrogenic segmental instability, which can lead to early adjacent segment disease requiring further surgical intervention (Amundsen et al., 2000).

In cases where patients exhibit extensive stenosis above a slipped segment, simultaneous decompression of the adjacent segment to the intervertebral fusion site is often necessary as a prophylactic measure; however, the optimal surgical strategy remains undetermined. Some surgeons advocate for performing laminectomy above the fused level (Smorgick et al., 2013), while others express concerns that additional decompression surgery combined with lumbar intervertebral fusion may exacerbate adjacent segment degeneration (Matsumoto et al., 2019). The high prevalence of ASD has facilitated innovations in hybrid surgical techniques, such as the topping-off technique, with studies indicating that its clinical outcomes are comparable to those of patients in intervertebral fusion (Sears et al., 2021; Wang et al., 2023; Okuda et al., 2018). In addition, it was challenging to stabilize the stenotic segments on the premise of adequate decompression and the literature is controversial with regard to progressive degeneration at the decompression level (Gard et al., 2013; Matsumoto et al., 2019; Mesregah et al., 2022; Sun et al., 2019).

There are currently few biomechanical studies that have investigated the biomechanical effects of concomitant decompression adjacent to a fusion segment. Therefore, the purpose of our study was to examine the biomechanical changes in the upper adjacent segment resulting from varying degrees of facet joint destruction in patients undergoing both intervertebral fusion and decompression surgery.

## 2 Methods

### 2.1 Finite element model development of lumbar spine

A young male volunteer (26 years old) with no history of lumbar spinal diseases underwent computed tomography (CT) scanning. The participant provided informed written consent for this study. CT images of the lumbar spine were imported into Mimics (Materialise NV, Leuven, Belgium) to construct a three-dimensional model. Geomagic Studio (Geomagic Inc., NC, USA) was utilized to reduce noise, remove spikes, smooth surfaces, and create patches and grids for meshing. The intervertebral discs were modeled in SolidWorks (SolidWorks Inc., MA, USA). The model was meshed in Hypermesh (Altair Technologies Inc., MA, USA), and biomechanical evaluation was conducted using Abaqus/Standard (Simulia Inc., RI, USA).

**FIGURE 1:**
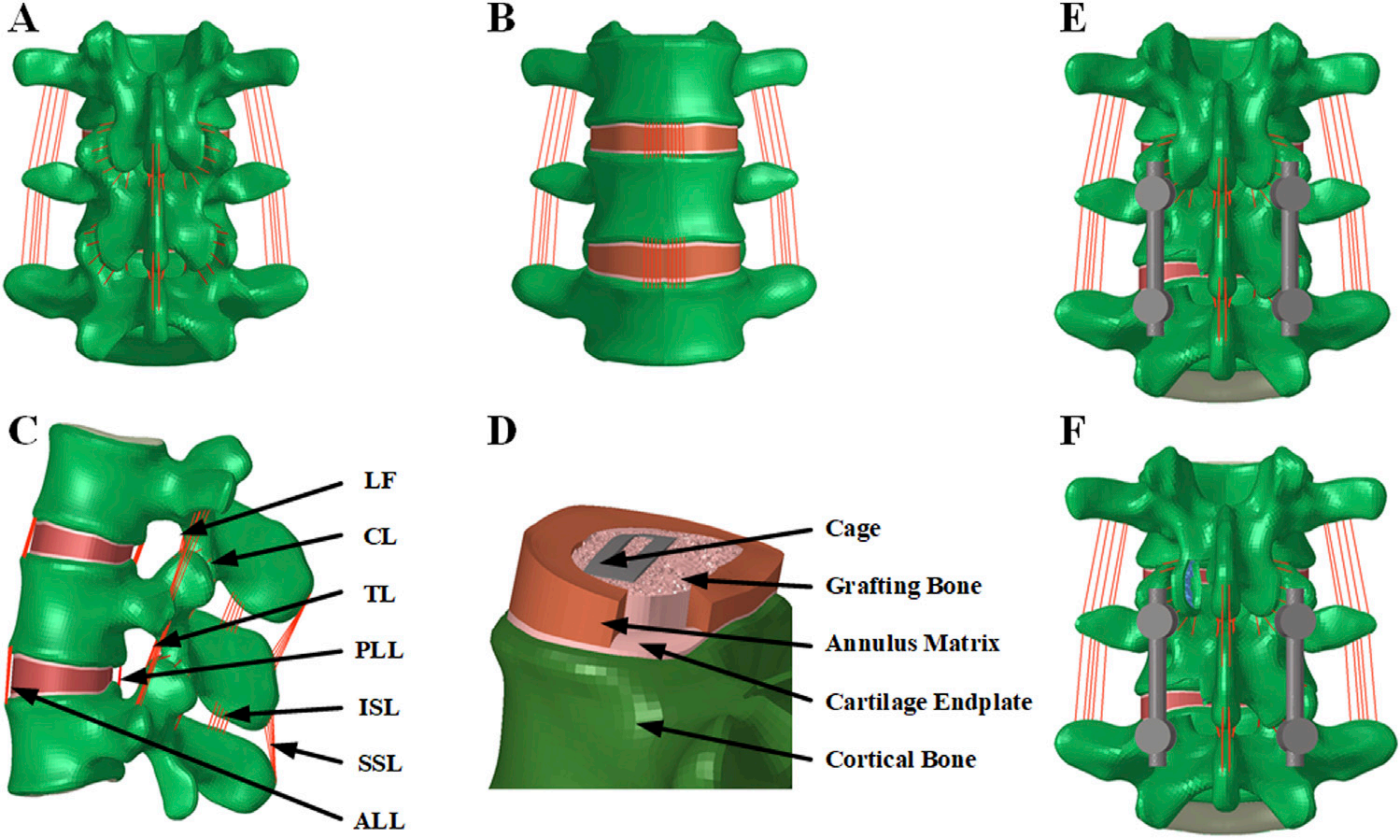
The intact finite element models of L3-L5. **(A)** Posterior view; **(B)** Anterior view; **(C)** Lateral view. **(D)** The intervertebral disc of L4/5 after TLIF surgery; **(E)** The posterior view of the finite element model after TLIF surgery; **(F)** The posterior view of the finite element model after TLIF and decompression surgery. ALL, anterior longitudinal ligament; PLL, posterior longitudinal ligament; CL, capsular ligament; LF, ligamentum flavum; ISL, interspinous ligament; IL, intertransverse ligaments; and SSL, supraspinal ligament.

As shown in Figure 1, the finite element (FE) model of the intact L3-L5 lumbar spine comprised three lumbar vertebrae, two intervertebral discs, and associated spinal ligaments. The vertebral body was divided into cancellous bone, cortical bone, and bone endplates. The cortical bone, endplate, annulus fibrosus matrix, and nucleus pulposus were all meshed using hexahedral elements. In contrast, the cancellous bone is discretized using tetrahedral elements. The thickness of the cortical bone is set to 1 mm. The facet joint surfaces were established using surface-to-surface contact elements, with an initial gap of 0.5 mm assumed between the interface elements and a friction coefficient of 0. The inferior endplate of L5 were restricted in six degrees of freedom. Each intervertebral disc consisted of the nucleus pulposus, annulus fibrosus, and superior and inferior cartilage endplates. The ligaments included in the lumbar FE model were the anterior longitudinal ligament (ALL), posterior longitudinal ligament (PLL), capsular ligament (CL), ligamentum flavum (LF), interspinous ligament (ISL), intertransverse ligaments (IL), and supraspinal ligament (SSL). Three-dimensional truss elements with no compression were employed to simulate the ligaments. The material properties were based on previous studies (Table 1) (Denozière and Ku, 2006; Kang et al., 2017; Kim et al., 2015). In total, 393,490 nodes and 960,415 elements were used for the developed intact spine model.

**TABLE 1:** Material properties of the finite element model.

Component	Young’s modulus (MPa)	Poisson’s ratio	Cross-section area (mm^2^)
Cortical bone	12000	0.3	-
Cancellous bone	100	0.2	-
Bone endplate	12000	0.3	-
Cartilage endplate	25	0.25	-
Annulus matrix	4.2	0.45	-
Nucleus pulposus	1	0.5	-
Anterior longitudinal ligament	7.8	0.3	63.7
Posterior longitudinal ligament	10	0.3	14.4
Capsular ligament	7.5	0.3	30
Ligamentum flavum	15	0.3	40
Interspinous ligament	10	0.3	26
Supraspinal ligament	8	0.3	23
Transverse ligament	10	0.3	1.8
Fusion cage	3600	0.3	-
Screws and rods	110000	0.3	-

### 2.2 FE model of L4-L5 intervertebral fusion and L3-L4 decompression procedures

The FE model with transforaminal lumbar interbody fusion (TLIF) at L4-L5 and decompression at L3-L4 were developed. To simulate the TLIF procedure, the left L4-5 facet joint and ligamentum flavum were completely excised. The intervertebral disc was removed and replaced with a single PEEK cage filled with cancellous bone, which was bonded to the vertebral bodies using a contact condition. Bilateral pedicle screw fixation was then added to L4-L5, with screws measuring 45 mm in length and 6.5 mm in diameter. The coefficient of friction between the implants and bone are 0.8. For the decompression procedure, a hemi-laminectomy was performed on the left side, along with the removal of part of the ligamentum flavum. However, the posterior ligamentous system, including the supraspinous and interspinous ligaments, was preserved to minimize disruption of biomechanical integrity.

Sixteen models were simulated in this study:

(1) the intact model without surgery (I),
(2) L4-L5 intervertebral fusion model (F),
(3) L3-L4 hemi-laminectomy and L4-L5 intervertebral fusion were both performed on the left side (DF);
(4) L3-L4 hemi-laminectomy was performed on the left side and L4-L5 intervertebral fusion was performed on the contralateral side (cDF).

Based on DF model, six scenarios were simulated according to the extent and degrees of FJV:

(5) left mild violation (LMV),
(6) left severe violation (LSV),
(7) right mild violation (RMV),
(8) right severe violation (RSV),
(9) bilateral mild violation (BMV),
(10) bilateral severe violation (BSV).

Based on cDF model, six scenarios were also simulated:

(11) left mild violation (cLMV),
(12) left severe violation (cLSV),
(13) right mild violation (cRMV),
(14) right severe violation (cRSV),
(15) bilateral mild violation (cBMV), and
(16) bilateral severe violation (cBSV).

**FIGURE 2:**
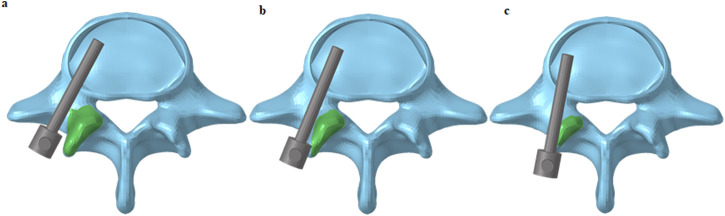
Criteria for grading violation of facet joint. **(a)** Screws were not in the facet joint and did not encroach the facet joint; **(b)** Screws encroached the lateral facet but did not enter the facet joint; **(c)** Screws passed through the articular surface of the facet joint.

Facet joint violation was simulated based on Babu’s grading system (Babu et al., 2012). In the mild violation model, the screws encroached on the lateral facet without entering the facet joint, whereas in the severe model, the screws penetrated the articular surface of the facet joint (Figure 2).

### 2.3 Boundary and loading conditions

The nodes on the inferior surfaces of L5 were constrained in all directions. An axial follower load of 400 N, combined with a pure moment of 10 Nm, was applied to the superior surface of L3 to simulate flexion, extension, lateral bending, and axial rotation. To produce the follower load, truss elements were created along the curved axis of the lumbar spine. The nodes of the truss elements were coupled to the intermediate nodes of each endplate surface. The follower load was applied to each segment through the truss elements. The ROM of L3-4 vertebrae and IDP in the L3/4 intervertebral discs were measured and compared across these surgical constructs. The ROMs at L3-L4 and L4-L5 of the intact FE model under 400 N compressive follower load and 10 Nm moment were quantified and compared with previous literature for validation.

## 3 Results

### 3.1 Model validation

**FIGURE 3:**
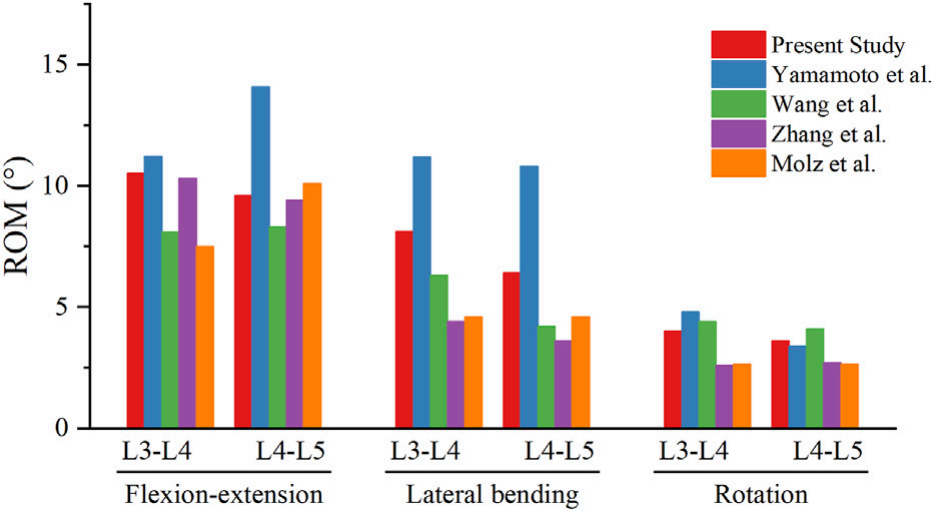
Comparison of range of motion between the current intact model and previous studies.

The results of the L3-4 and L4-5 ROMs were consistent with previous FE and *in vitro* studies (Figure 3) (Wang et al., 2019; Yamamoto et al., 1989), thereby validating the model. The ROM and IDP were analyzed and compared to elucidate the biomechanical effects of FJV and decompression at the supra-adjacent segment.

### 3.2 Comparison of ROM and IDP among models

The changes in ROM and IDP were expressed as percentage changes from those of the intact model. In the eight models, the ROM and IDP during flexion, extension, lateral bending, and rotation were found to be increased compared to the intact model.

### 3.3 Biomechanical changes of L3-L4 in the decompression model

**FIGURE 4:**
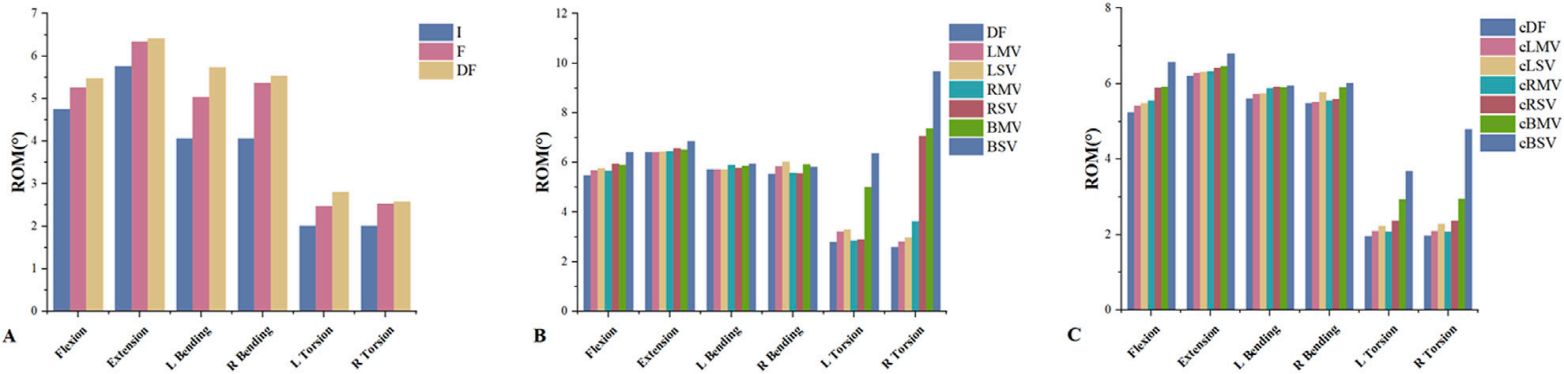
**(A)** The ROM in the I, F and DF models under loading conditions of flexion, extension, left bending, right bending, left torsion and right torsion. **(B)** The ROM in the DF, LMV, LSV, RMV, RSV, BMV and BSV models under the six loading conditions. **(C)** The ROM in the cDF, cLMV, cLSV, cRMV, cRSV, cBMV and cBSV models under the six loading conditions. ROM, range of motion. I, the intact model without surgery. F, L4-L5 intervertebral fusion model. DF, L3-L4 hemi-laminectomy and L4-L5 intervertebral fusion were both performed on the left side. LMV, left mild violation. LSV, left severe violation. RMV, right mild violation. RSV, right severe violation. BMV, bilateral mild violation. BSV, bilateral severe violation. cDF, L3-L4 hemi-laminectomy was performed on the left side and L4-L5 intervertebral fusion was performed on the contralateral side. cLMV, left mild violation. cLSV, left severe violation. cRMV, right mild violation. cRSV), right severe violation. cBMV, bilateral mild violation. cBSV, bilateral severe violation.

L3-L4 decompression resulted in an increased ROM under six loading conditions (Figure 4). A significant increase in ROM was observed in the DF model compared to the F model, with changes of 4.13%, 1.25%, 13.67%, 3.13%, 13.28%, and 1.90% for flexion, extension, left bending, right bending, left torsion, and right torsion, respectively. The decompression model also exhibited increased IDP under the same six loading conditions. The DF model showed increases of 19.95%, 0.24%, 9.58%, 5.81%, 3.48%, and 5.59% in IDP compared to the F model for flexion, extension, left bending, right bending, left torsion, and right torsion, respectively.

### 3.4 Biomechanical changes of L3-L4 in the FJV model

**FIGURE 5:**
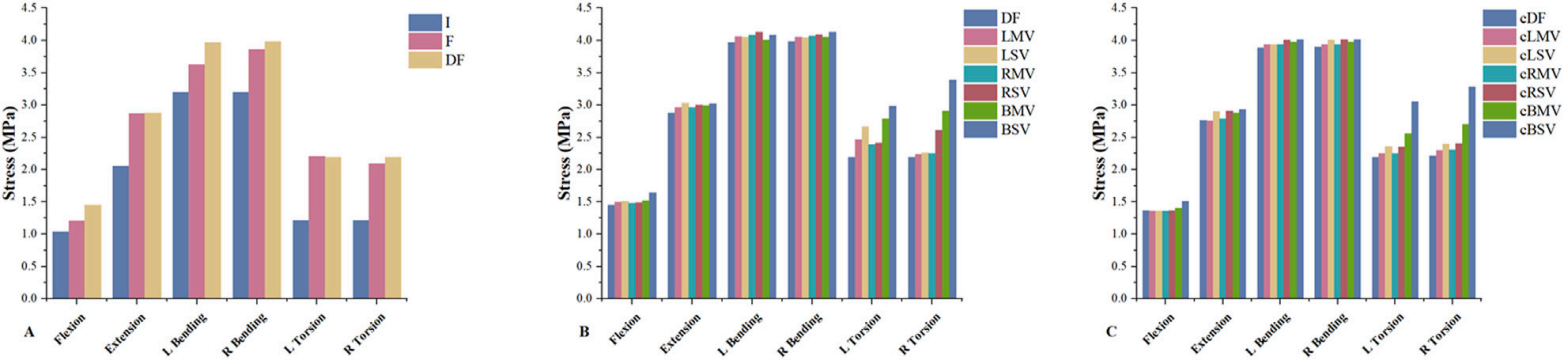
**(A)** The IDP at L3-L4 in the I, F and DF models under loading conditions of flexion, extension, left bending, right bending, left torsion and right torsion. **(B)** The IDP at L3-L4 in the DF, LMV, LSV, RMV, RSV, BMV and BSV models under the six loading conditions. **(C)** The IDP at L3-L4 in the cDF, cLMV, cLSV, cRMV, cRSV, cBMV and cBSV models under the six loading conditions. IDP, intradiscal pressure. I, the intact model without surgery. F, L4-L5 intervertebral fusion model. DF, L3-L4 hemi-laminectomy and L4-L5 intervertebral fusion were both performed on the left side. cDF, L3-L4 hemi-laminectomy was performed on the left side and L4-L5 intervertebral fusion was performed on the contralateral side. LMV, left mild violation. LSV, left severe violation. RMV, right mild violation. RSV, right severe violation. BMV, bilateral mild violation. BSV, bilateral severe violation. cLMV, left mild violation. cLSV, left severe violation. cRMV, right mild violation. cRSV), right severe violation. cBMV, bilateral mild violation. cBSV, bilateral severe violation.

The presence of facet joint violation resulted in an increase in ROM (Figure 5). Among the six motions, the most significant increases in ROM were observed in the right and left rotation models. Compared to the DF model, the ROM in the LMV, LSV, RMV, RSV, BMV, and BSV models under left torsion increased by 15.15%, 18.3%, 1.75%, 3.11%, 79.09%, and 127.45%, respectively. Similarly, under right torsion, the increases were 9.31%, 15.16%, 40.87%, 173.71%, 185.73%, and 275.11%, respectively. Compared to the cDF model, the ROM in the cLMV, cLSV, cRMV, cRSV, cBMV, and cBSV models under left torsion increased by 6.68%, 13.66%, 5.81%, 20.39%, 49.39%, and 138.12%, respectively. Similarly, under right torsion, the increases were 6.50%, 15.48%, 5.69%, 19.70%, 49.49%, and 143.05%, respectively.

Under torsional moments, IDP was also significantly affected by facet joint violation, with IDP increasing alongside the degree of violation. Compared to the DF model, IDP in the LMV, LSV, RMV, RSV, BMV, and BSV models under left torsion increased by 12.38%, 42.82%, 8.82%, 9.99%, 2.85%, and 16.54%, respectively. For right torsion, the increases were 1.91%, 2.48%, 2.38%, 19.02%, 33.84%, and 57.93%, respectively. Compared to the cDF model, IDP in the cLMV, cLSV, cRMV, cRSV, cBMV, and cBSV models under left torsion increased by 2.74%, 7.43%, 2.78%, 7.34%, 16.46%, and 39.35%, respectively. For right torsion, the increases were 4.12%, 8.46%, 4.34%, 8.87%, 22.17%, and 48.37%, respectively.

**FIGURE 6:**
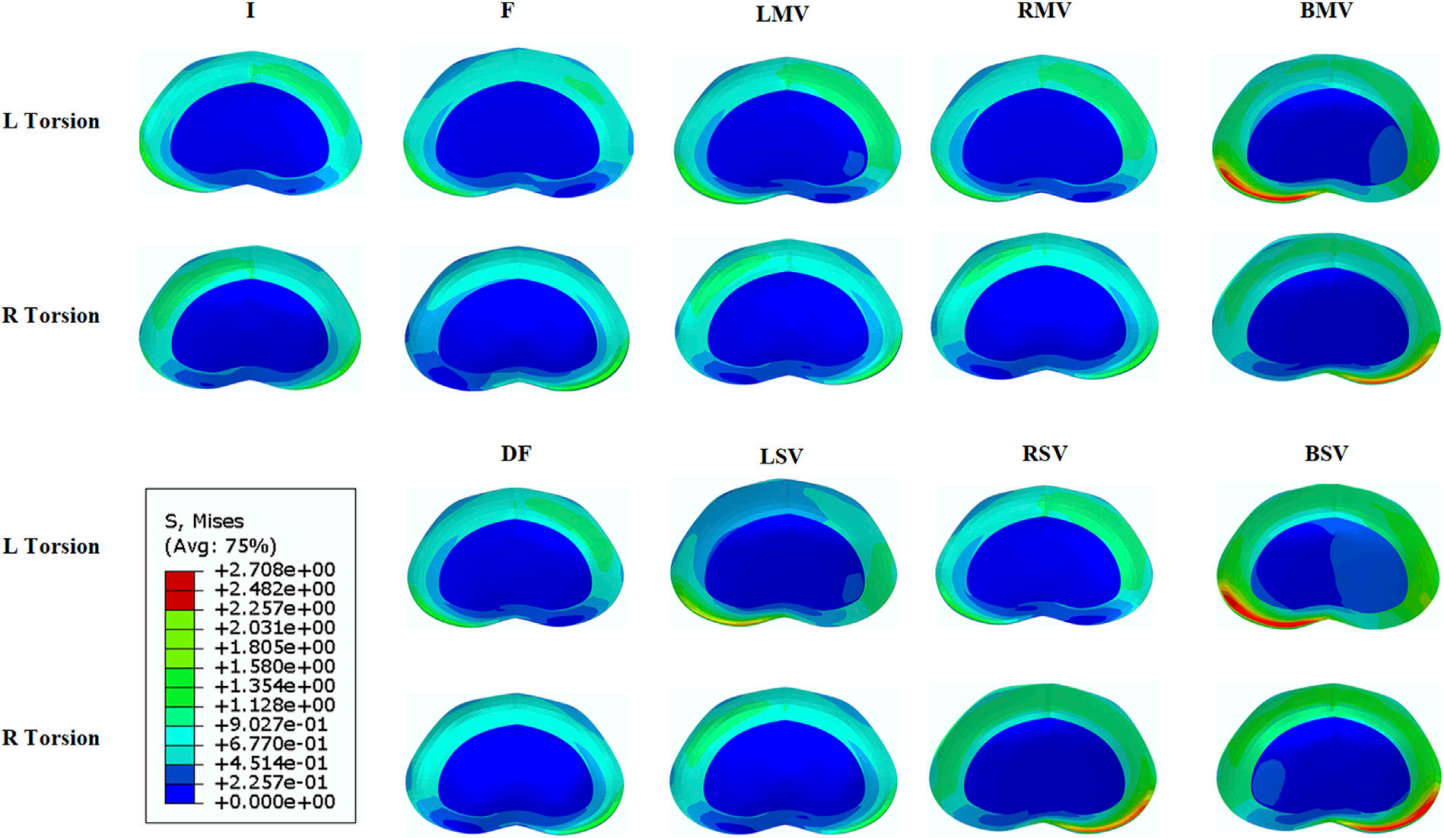
Von Mises stress distribution at L3/4 with the L3-L4 hemi-laminectomy and L4-L5 intervertebral fusion were both performed on the left side. I, the intact model without surgery. F, L4-L5 intervertebral fusion model. DF, L3-L4 hemi-laminectomy and L4-L5 intervertebral fusion were both performed on the left side. LMV, left mild violation. LSV, left severe violation. RMV, right mild violation. RSV, right severe violation. BMV, bilateral mild violation. BSV, bilateral severe violation.

**FIGURE 7:**
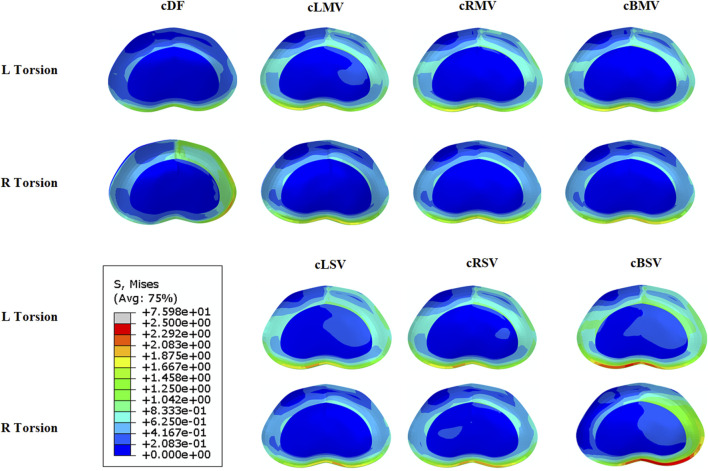
Von Mises stress distribution at L3/4 with the L3-L4 hemi-laminectomy was performed on the left side and L4-L5 intervertebral fusion was performed on the contralateral side. cDF, L3-L4 hemi-laminectomy was performed on the left side and L4-L5 intervertebral fusion was performed on the contralateral side. cLMV, left mild violation. cLSV, left severe violation. cRMV, right mild violation. cRSV), right severe violation. cBMV, bilateral mild violation. cBSV, bilateral severe violation.

Left facet joint violation resulted in greater ROM and IDP during left rotation, while right facet joint violation produced similar effects in right rotation models. The models with bilateral facet joint violation exhibited the most significant increases in both ROM and IDP. Figures 6, 7 illustrates the stress distribution on the intervertebral disc of L3/4 in the models with left and right torsion.

## 4 Discussion

Although satisfactory clinical outcomes have been reported for lumbar intervertebral fusion, concerns regarding ASD persist, as it is a long-term complication that requires careful monitoring (Maragkos et al., 2020; Zhong et al., 2017). Risk factors for ASD include FJV, fusion length, sagittal alignment, and decompression outside the fusion construct (Kim et al., 2016; Maragkos et al., 2020; Mesregah et al., 2022). Among the risk factors for ASD, facet joint violation is most closely associated with the surgeon’s skill level and surgical details. Both unilateral and bilateral violations have been shown to worsen patient outcomes, as evidenced by higher Visual Analog Scale (VAS) scores and Oswestry Disability Index (ODI) scores (Jia et al., 2018; Zhao et al., 2023), and the postoperative biomechanical effects on the lumbar spine correlate with the extent of violation (Zhao et al., 2020). Detailed reports exist regarding the clinical outcomes and biomechanical effects of FJV in both single-level and multi-level lumbar fusion surgeries (Wang et al., 2015; Tannous et al., 2017; Wangsawatwong et al., 2023). However, when the two surgical procedures are combined, the biomechanical effects of FJV on the lumbar spine may differ. FE analysis serves as an effective, noninvasive method to evaluate biomechanical changes post-operation. This study generated and validated FE models to investigate the biomechanical effects of FJV following transforaminal lumbar intervertebral fusion adjacent to the decompression segment, assessing the biomechanical variation in adjacent segments due to different grades of FJV. The findings indicated that flexion produced the greatest increase in IDP under decompression surgery, while the two torsional movements following intervertebral fusion and decompression surgery led to significant increases in both ROM and IDP. Increases in IDP and ROM were more pronounced in rotational movements directed towards the violation side. Bilateral facet joint violation significantly diminished lumbar spine stability and heightened stress concentration.

For degenerative lumbar diseases, lumbar intervertebral fusion is recommended for conditions involving segmental instability, such as degenerative lumbar spondylolisthesis (DLS), while decompression is suggested for conditions like lumbar spinal stenosis (LLS) without instability. In patients at risk for extensive stenosis adjacent to DLS, both decompressive laminectomy and lumbar intervertebral fusion may be necessary. Previous studies have yielded conflicting results regarding ASD following decompression adjacent to the fusion segment (Gard et al., 2013; Matsumoto et al., 2019; Miyagi et al., 2013). Matsumoto et al. (Matsumoto et al., 2019) found that concomitant decompression exacerbated disc degeneration but did not induce segmental instability, while Miyagi et al. (Miyagi et al., 2013) reported a higher incidence of ASD with additional decompression, suggesting that compromising the posterior complex integrity increases segmental instability risk. The biomechanical mechanisms underlying these phenomena remain unclear. The most frequently operated levels in lumbar spine surgery are L4-S1 due to high incidences of degenerative disc disease, spondylolisthesis, and spinal stenosis. However, the L5-S1 segment behaves uniquely due to its articulation with the sacrum, which has a high load-bearing function and limited mobility compared to other lumbar segments. L3-L5 avoids complexities associated with the transitional biomechanics of L5-S1. As a result, L3-L5 often serves as the first adjacent motion segment, making it a prime region for studying early degenerative changes, increased motion, and stress redistribution that may contribute to ASD. In this study, even with preservation of the posterior ligamentous system, the DF model exhibited increased ROM and IDP at the adjacent segment compared to the F model, particularly during flexion and left torsion moments, potentially accelerating intervertebral disc and facet joint degeneration and progression of ASD. Therefore, caution is warranted in performing decompression surgery near the fusion segment, balancing surgical benefits against the heightened risk of adjacent segment degeneration.

The surgical procedure for intervertebral fusion may inadvertently injure facet joints, resulting in FJV and functional impairment, which are often underestimated complications. As FJV is not a component of the standardized surgical procedure but rather an accidental injury caused by various factors during surgery, its severity cannot be subjectively controlled. This variation is possibly related to differences in populations, pathologies, or fusion procedures. It is reported that robot-assisted surgery had 69% and 92% significantly less likelihood of complications and proximal facet joint violation respectively compared to the free-hand technique (Fatima et al., 2021). It is a benefit but relies on the accuracy of image acquisition and registration. In any cases, it is crucial to carefully protect the facet joint when using any technique for pedicle screw insertion. Research findings indicate that the severity of FJV is closely associated with postoperative clinical outcomes. Patients with moderate and severe FJV have significantly higher VAS scores for lower back pain and ODI scores compared to those without FJV (Jia et al., 2018; Zhao et al., 2023). Moreover, patients with bilateral involvement exhibit worse VAS and ODI scores than those with unilateral involvement (Cardoso et al., 2008). Although researchers currently employ differing definitions and grading criteria for FJV, common grading systems typically range from no invasion or minimal joint surface invasion to complete destruction of the joint surface. Therefore, this study categorizes FJV into mild and severe grades based on the extent of preservation of the facet joint mechanical structure, while also differentiating between left-side and right-side invasions. The incidence of FJV varies widely despite advances in robotic-assisted or fluoroscopy-guided techniques, primarily due to a lack of awareness regarding protective measures during surgery (Xu et al., 2020). Violations at the supra-adjacent segment during pedicle screw placement alter load-bearing capabilities, accelerating facet joint degeneration, stiffness, rigidity, and osteoarthritis, ultimately leading to adjacent segment degeneration. Cardoso et al. (Cardoso et al., 2008) conducted a cadaveric study indicating that supra-adjacent FJV results in torsional instability post-surgery. Kim et al. (Kim et al., 2012) further supported this by demonstrating significant increases in facet contact force and IDP under extension and torsion moments in their finite element model. Our results align with previous studies showing that FJV significantly increases ROM and IDP at the supra-adjacent segment, correlating with the extent and severity of violation. Bilateral facet joint violation amplified biomechanical effects on the lumbar spine, resulting in greater stress concentration and an elevated risk of degeneration. Furthermore, the combination of facet joint violation and decompression had a synergistically adverse impact on adjacent segment degeneration, particularly when both occurred on the ipsilateral side.

This study utilized finite element analysis to explore the biomechanical effects of intervertebral fusion surgery combined with adjacent decompression on the lumbar spine, revealing synergistic effects between the two procedures. These findings provide a reference for surgical decision-making and underscore the importance of avoiding FJV, especially bilateral violations. In the case of unilateral facet joint involvement alone, there is no necessity to extend the fusion segments. However, concurrent bilateral facet joint involvement can significantly compromise spinal stability, particularly during rotational movements. Furthermore, extensive damage may increase the likelihood of developing facet joint arthritis, thereby warranting consideration of extending the instrumentation segment cranially. When decompression surgery is performed at a level adjacent to the instrumented segment, the increased risk of instability associated with combining decompression and instrumentation should be taken into account, especially when bilateral facet joint involvement is present, under which circumstances extending the instrumentation segment may be a more favorable option.

There were several limitations in our study. First, the finite element model was based on the structural the structural CT imaging of a single individual, which may not completely represent the mechanical outcomes for all patients. Second, excluding thoracic vertebrae and structures above may have amplified the surgical impact on the lumbar spine. Third, only ideal surgical conditions were considered, whereas actual outcomes depend on the surgeon’s expertise and the patient’s individual condition. Finally, various risk factors are associated with the development of ASD. However, only the impact of FJV on the development of ASD was analyzed in this study, while the roles of various other factors were ignored.

## 5 Conclusion

The present study evaluated the biomechanical effects of facet joint violation after transforaminal lumbar intervertebral fusion adjacent to a decompression segment. Our findings suggested that both concomitant adjacent decompression and FJV increased the IDP and ROM at the adjacent segment. Moreover, the combination of these factors might act synergistically, increasing the risk of adjacent segment degeneration, especially when violation occur red bilaterally or when the violation and decompression were on the ipsilateral side, amplifying their adverse impact on spinal stability.

## Data Availability

The original contributions presented in the study are included in the article/supplementary material, further inquiries can be directed to the corresponding authors.
